# Discovery of Novel Antimicrobial-Active Compounds and Their Analogues by In Silico Small Chemical Screening Targeting *Staphylococcus aureus* MurB

**DOI:** 10.3390/molecules30071477

**Published:** 2025-03-26

**Authors:** Saya Okubo, Shoki Hirose, Shunsuke Aoki

**Affiliations:** Department of Bioscience and Bioinformatics, Graduate School of Computer Science and Systems Engineering, Kyushu Institute of Technology, Iizuka 820-8502, Japans.hirose1521@gmail.com (S.H.)

**Keywords:** *Staphylococcus*, MurB, *SBDS*, virtual screening, MD simulation, in vitro experiment, enantiomer

## Abstract

Methicillin-resistant *Staphylococcus aureus* is a serious problem in healthcare due to its lethal severe infections and resistance to most antimicrobial agents. The number of new approved antimicrobial agents is declining, and combined with the spread of drug-resistant bacteria, it is predicted that effective antimicrobial agents against multidrug-resistant bacteria will be exhausted. We conducted in silico and in vitro discovery of novel antimicrobial small molecules targeting the SaMurB enzyme involved in cell wall synthesis in *Staphylococcus aureus* (*S. aureus*). We performed hierarchical structure-based drug screenings to identify compounds and their analogues using a library of approximately 1.3 million compound structures. In vitro experiments with *Staphylococcus epidermidis* (*S. epidermidis*) identified three compounds (SH5, SHa6, and SHa13) that exhibit antibacterial activity. These three compounds do not have toxicity against human-derived cells. SHa13 exhibited remarkable activity (IC_50_ value =1.64 ± 0.01 µM). The active compound was predicted to bind to the active site of SaMurB by forming a hydrogen bond with Arg188 in both R and S bodies. These data provide a starting point for the development of novel cell wall synthesis inhibitors as antimicrobial agents targeting SaMurB.

## 1. Introduction

*Staphylococcus aureus* (*S. aureus*) is a Gram-positive bacterium commonly found on the human skin and in the nasal cavity. In elderly and postoperative patients with weakened immune systems, *S. aureus* can cause severe symptoms such as pneumonia and sepsis [1,2,3,4,5,6,7]. Infections caused by methicillin-resistant *S. aureus* (MRSA) are complicated to treat because of its antibiotic resistance [8,9]. Vancomycin is commonly used to treat MRSA infections; however, vancomycin-resistant *S. aureus* (VRSA) has already emerged [10]. Although only nosocomial MRSA (HA-MRSA) was prevalent, community-acquired MRSA (CA-MRSA) has been identified, and the outflow of multidrug-resistant *S. aureus* outside hospitals is a severe problem [11,12]. The global spread of multidrug-resistant *S. aureus* is ongoing and is anticipated to become a major societal problem that will strain medical practices in the future. Despite the foreseeable future crisis of drug-resistant bacteria, the speed of development and approval of new antimicrobial agents is inadequate, and the depletion of effective agents is imminent [13]. In contrast to conventional experimental drug development methods using in vitro and in vivo methods, an efficient approach using in silico computational drug screening is expected to increase the speed of drug discovery and development [14,15]. The peptidoglycan that forms the *S. aureus* cell wall consists mainly of a cross-linked structure of *N*-acetylglucosamine and *N*-acetylmuraminic acid [16,17]. UDP-*N*-acetylenolpyruvylglucosamine reductase (MurB) is an essential enzyme involved in the peptidoglycan synthesis pathway of *S. aureus*. In the peptidoglycan synthesis pathway of *S. aureus*, the MurA enzyme initially transfers enolpyruvate (EP) from phosphoenolpyruvate (PEP) to UDP-*N*-acetylglucosamine to produce EP-UDP-*N*-acetylglucosamine [18,19]. The MurB enzyme then converts EP-UDP-*N*-acetylglucosamine to EP-UDP-*N*-acetylmuramic acid in a NADPH-dependent manner [20,21]. Furthermore, MurC-F catalyzes multiple small molecule linkage reactions to EP-UDP-*N*-acetylmuraminic acid, followed by various other enzymatic reactions that ultimately form peptidoglycan molecules. MurB is essential for the survival of *S. aureus* because deficiency of peptidoglycan leads to inhibition of bacterial cell wall synthesis, suppression of bacterial growth, and lysis due to loss of resistance to osmotic pressure. An inhibitor of *S. aureus* against MurB was identified and reported to have antibacterial activity [22,23]. However, no antimicrobial medical drug targeting MurB is approved, and no MurB-inhibiting small molecule compounds are developed in the clinical trial stage [24]. Structure-based drug screening (SBDS) is one of the most effective computational methods for new drug discovery. Successful identifications of antimicrobial-active compounds for *S. aureus* and *Mycobacterium tuberculosis* by SBDS have been reported [25,26,27,28,29,30]. Docking simulation tools used in SBDS include DOCK [31], GOLD [32], GLIDE [33], AutoDock Vina [34], rDock [35], FRED [36], SMINA [37], and others. The hierarchical SBDS system combined with multiple docking simulation tools enables efficient identification of pharmacologically active compounds with specific binding capacity to their targets [22,23,24,25,26,27]. In this study, we targeted the active site pocket structure of MurB and successfully identified novel antibacterial active compounds against *S. aureus* using a three-step in silico SBDS approach. Furthermore, molecular dynamics simulations showed stable binding of the antimicrobial-active compounds to MurB. We also identified several novel antibacterial compounds against *S. aureus* by searching for analogues and obtained useful information for drug development targeting MurB in structure–activity relationships.

## 2. Results

### 2.1. Hierarchical In Silico SBDS

**Figure 1:**
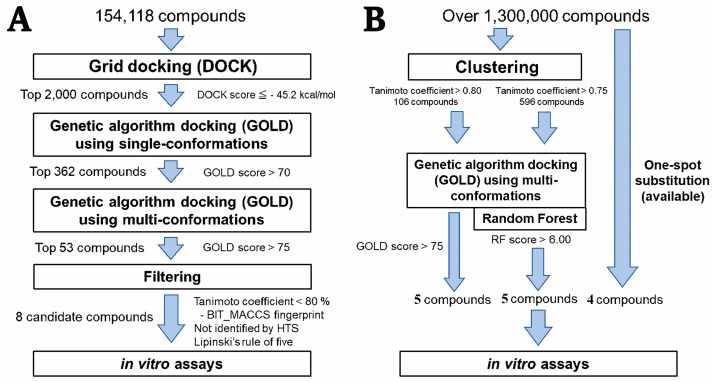
In silico compound screening pathway. Hierarchical SBDS pathway (**A**). Screening pathway for SH5 analogues (**B**).

Hierarchical in silico SBDS was performed on the *S. aureus* MurB (SaMurB) crystal structure data. The screening system consisted of three steps (Figure 1A). The DOCK program (UCSF DOCK version 6.4) was used for rigid docking in the first screening, and the GOLD program (CCDC GOLD suite version 5.2.2) was used for flexible docking by genetic algorithm in the second and third screenings. The compound structure libraries in the second and third screenings contained single and multiple conformation compound structures, respectively. Each docking simulation was performed for the active site pocket of SaMurB (center coordinates: x = 157.87, y = 155.04, z = 152.24). In the first screening, the binding affinity of all compounds to SaMurB was calculated using a compound structure library (154,118 compounds), and the top 2000 compounds (binding free energy < −45.18 kcal/mol, top 1.3%) of the top 2000 compounds were selected. In the second screening, the structure library consisting of the 2000 compounds was used to calculate the Gold score by GOLD. The top 362 compounds (Gold score > 70.00) were selected, and a library of 1060 compound structures was constructed by generating a maximum of 10 different conformations per compound. In the third screening, docking simulations with GOLD were performed under the same conditions as the second screening. After calculating the average Gold score for each compound, the top 53 compounds (Gold score > 75.00) were selected. Fingerprints based on the BIT_MACCS Key [38] were calculated for those 53 compounds and classified based on 90% similarity using the Tanimoto coefficient. The compound with the highest Gold score in the same cluster was designated as the representative of the cluster. The eight compounds that did not violate more than three conditions in Lipinski’s rule [39] of five were selected as the final candidates (SH1-SH3, [*R*]-SH4, [*R*]-SH5, and SH6-SH8). The PubChem [40] database revealed no in vitro antibacterial assay data against *S. aureus* using these candidate compounds (Table S1).

### 2.2. Growth Inhibition Activity of Compounds for Staphylococcus Bacteria

**Figure 2:**
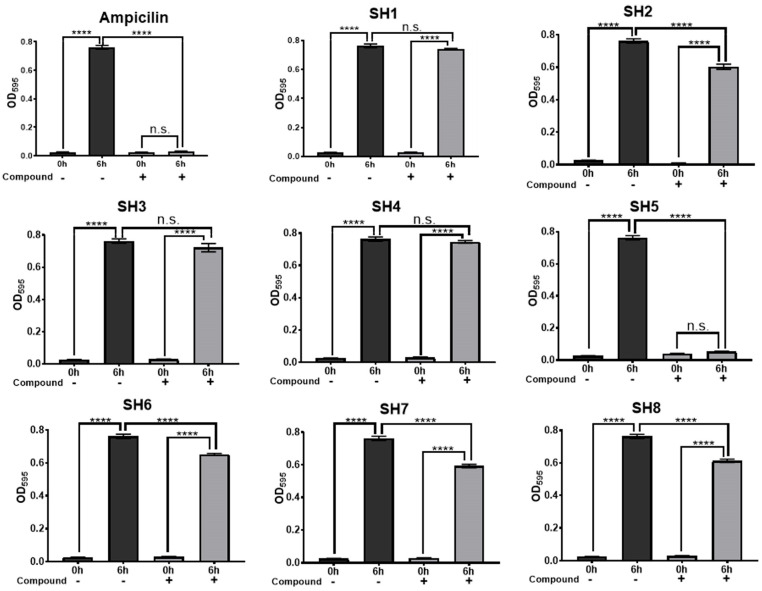
Growth inhibitory activity of compounds (SH1-SH3, [*R*,*S*]-SH4, [*R*,*S*]-SH5, SH6-SH8) against *S. epidermidis.* A total of 100 µM ampicillin (ABPC) was used as the positive control and 0.3% DMSO as the negative control. Compounds were added at a concentration of 100 µM. Each value represents the mean ± SEM of four independent experiments. (Tukey’s multiple comparison test: **** *p* < 0.0001, n.s. = not significant).

*Staphylococcus epidermidis* (*S. epidermidis*) was used to test the growth inhibitory effect of the compounds on staphylococci. The results of the growth inhibition assay against *S. epidermidis* using the candidate compounds SH1-SH3, (*R*,*S*)-SH4, (*R*,*S*)-SH5, SH6-SH8 are shown in Figure 2. Using BLAST [41] homology search, we confirmed that the amino acid sequence of SaMurB is similar to that of *S. epidermidis* MurB (84% identity, and 93% similarity). Among the eight candidate compounds, SH2, (*R*,*S*)-SH5, SH6, SH7, and SH8 significantly inhibited *S. epidermidis* growth compared to the negative control, DMSO (Figure 2). Compared to DMSO conditions, SH2, (*R*,*S*)-SH5, SH6, SH7, and SH8 showed growth inhibition rates of 19.0%, 98.4%, 15.3%, 23.5%, and 20.8%, respectively.

### 2.3. Screening for Analogues of Active Compounds

We searched for analogues based on the structure of (*R*)-SH5, which showed the strongest antibacterial activity against *S. epidermidis* (Figure 1B). Compound data with more than 75% similarity (BIT_MACCS Key) to (*R*)-SH5 were obtained from the ChemBridge compound structure data library, which has approximately 1.3 million compounds. After structural optimization by MOE, approximately 10 conformations (top 10 most energy-stable conformations) were generated per compound, and the Gold score was calculated by docking simulation using GOLD. The top five compounds with the highest average GOLD score were selected. Rescoring was performed with the RFscoreVS v2 [42] program using the random forest algorithm with docking pose data predicted by GOLD. The top five resulting compounds (RF score > 6.09) were selected. Compounds that differ in only one substructure of (*R*)-SH5 or have more than 90% similarity to (*R*)-SH5 were selected (four compounds). Finally, 14 SH5 analogues ([*R*]-SHa1-[*R*]-SHa14) were obtained (Table S2, Figure S1).

### 2.4. In Vitro Growth Inhibition Assay of SH5 Analogues Against S. epidermidis

**Figure 3:**
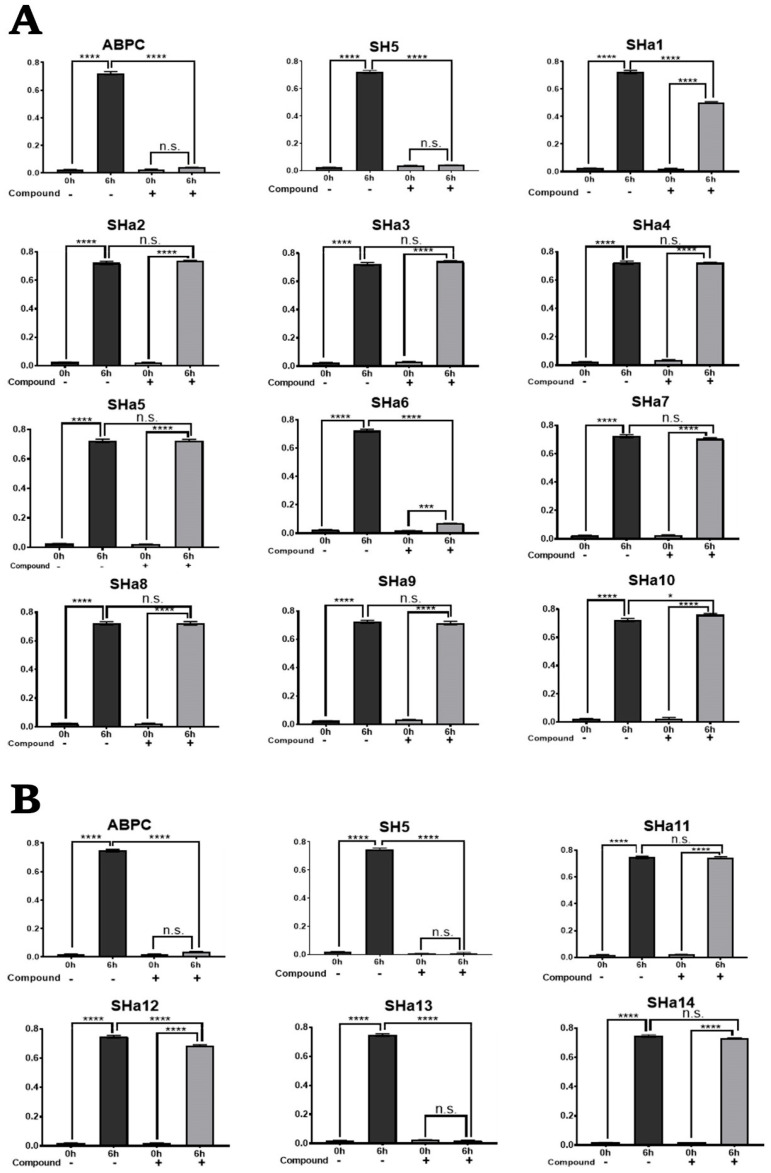
Growth inhibitory effect of SH5 analogues ([*R*,*S*]-SHa1-SHa14) on *S. epidermidis*. Growth inhibition effect of (*R*,*S*)-SHa1-(*R*,*S*)-SHa10 (**A**) and (*R*,*S*)-SHa11-(*R*,*S*)-SHa14 (**B**). A total of 100 µM ampicillin (ABPC) was used as the positive control and 0.3% DMSO as the negative control. Compounds were added at a concentration of 100 µM. Each value represents the mean ± SEM of four independent experiments. (Tukey’s multiple comparison test: **** *p* < 0.0001, *** *p* < 0.0002, * *p* < 0.0332, n.s. = not significant).

The results of the growth inhibition assay of analogous compounds (*R*,*S*)-SHa1-(*R*,*S*)-SHa14 against *S. epidermidis* are shown in Figure 3. Among the fourteen compounds, (*R*,*S*)-SHa1, (*R*,*S*)-SHa6, (*R*,*S*)-SHa12, and (*R*,*S*)-SHa13 significantly inhibited the growth of *S. epidermidis* compared to DMSO conditions (Figure 3). Compared to DMSO conditions, (*R*,*S*)-SHa1, (*R*,*S*)-SHa6, (*R*,*S*)-SHa12, and (*R*,*S*)-SHa13 showed 31.0%, 92.6%, 8.4%, and 99.9% growth inhibition, respectively.

### 2.5. Dose-Dependent Growth Inhibition Assay Against S. epidermidis

**Figure 4:**
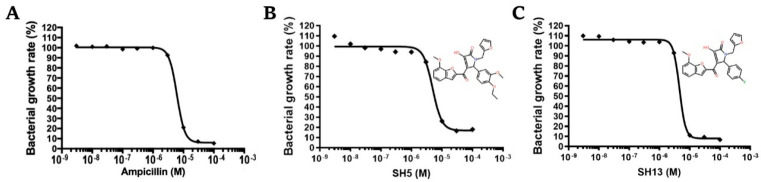
Dose-dependent effect of growth inhibition of (*R*,*S*)-SH5 and (*R*,*S*)-SHa13 against *S. epidermidis*. Growth inhibition curve for ampicillin (**A**). Growth inhibition curve for (*R*,*S*)-SH5 (**B**). Growth inhibition curve for (*R*,*S*)-SHa13 (**C**). Each plot represents the mean ± SEM of four independent experiments. We have conducted this experiment three times and obtained similar results in all cases.

The dose-dependent effects of the active compounds, (*R*,*S*)-SH5, (*R*,*S*)-SHa6, and (*R*,*S*)-SHa13, on the growth of *S. epidermidis* were investigated, and 50% growth inhibition concentrations (IC_50_ value) were calculated. The IC_50_ values for (*R*,*S*)-SH5 and (*R*,*S*)-SHa13 were 5.67 ± 0.51 µM and 1.64 ± 0.10 µM, respectively (Figure 4). The exact IC_50_ value for (*R*,*S*)-SHa6 could not be determined; however, the IC_50_ value was estimated to be less than 100 µM.

### 2.6. In Vitro Assay for Escherichia coli

**Figure 5:**
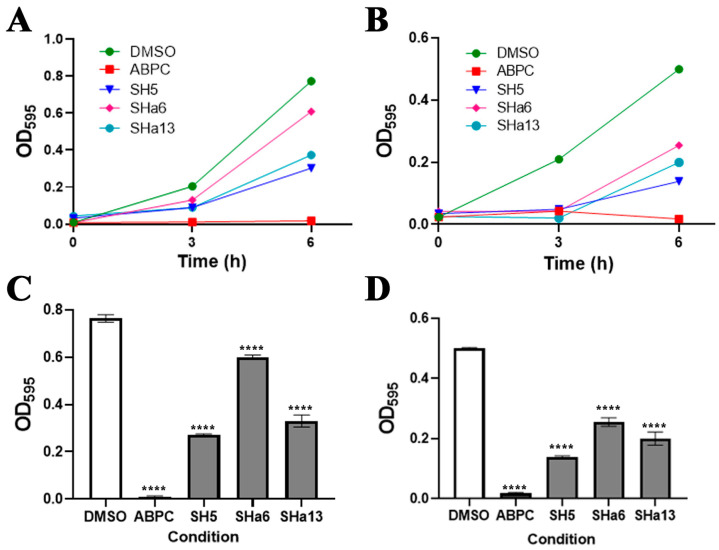
Growth inhibitory effect of active compounds on Gram-negative bacteria. Effect of the compound on the growth of *E. coli* BL21 strain (**A**,**C**). Effect of the compound on growth of *E. coli* JM109 strain (**B**,**D**). A total of 100 µM ampicillin (ABPC) was used as the positive control and 0.3% DMSO as the negative control. Compounds were added at a concentration of 100 µM. Each value represents the mean of four independent experiments (**A**,**B**). Each value represents the mean ± SEM of four independent experiments (**C**,**D**). We have conducted this experiment three times and obtained similar results in all cases. (Dunnett’stest: **** *p* < 0.0001).

The growth inhibitory effects of (*R*,*S*)-SH5, (*R*,*S*)-SHa6, and (*R*,*S*)-SHa13 for Gram-negative bacteria (*E. coli*), BL21, and JM109 strains were examined. BLAST analysis showed 21% amino acid sequence identity and 41% homology between SaMurB and *E. coli* MurB (PDBID: 2MBR). (*R*,*S*)-SH5, (*R*,*S*)-SHa6, and (*R*,*S*)-SHa13 showed weak to moderate antimicrobial activity against BL21 and JM109 strains (Figure 5). The growth inhibition rates of (*R*,*S*)-SH5 against BL21 and JM109 strains were 64.6% and 78.1%, respectively. The growth inhibition rates of (*R*,*S*)-SHa6 and (*R*,*S*)-SHa13 against BL21 and JM109 strains were 55.3% and 21.4%, 63.6% and 56.8%, respectively. Ampicillin at 100 µM exhibited a complete anti-bactericidal effect, while compounds (*R*,*S*)-SH5, (*R*,*S*)-SHa6, and (*R*,*S*)-SHa13 at the same concentration showed relatively weak antibacterial effects.

### 2.7. In Vitro Toxicity Assay for Human Cells

The toxicity of (*R*,*S*)-SH5, (*R*,*S*)-SHa6, and (*R*,*S*)-SHa13 against PC3 and HepG2 was examined. No significant differences in survival rates were observed in the (*R*,*S*)-SH5-added condition compared to the negative control DMSO condition, and (*R*,*S*)-SH5 was not toxic to both human cells. Survival rates of human cells under (*R*,*S*)-SHa6- and (*R*,*S*)-SHa13-added conditions were not significantly reduced compared to the negative control DMSO (Figure S2).

### 2.8. Prediction of Binding Mode of Active Compounds to SaMurB

To investigate the binding mode of the active compounds to SaMurB, we generated from 23 to a maximum of 267 conformations for (*R*,*S*)-SH5, (*R*,*S*)-SHa6, and (*R*,*S*)-SHa13 and performed docking simulations with GOLD. From the results, we ranked the Gold score for each compound and examined the binding mode of the compound with the top 10 Gold score. PLIF and LI [43] analysis were used to evaluate the interaction with SaMurB. The active compounds are all enantiomers with one chiral carbon, and the reagents used in the experiments are racemic. Therefore, the binding modes were predicted using the structures of the *R*- and *S*-body compounds (Table 1, Figure S9). The average Gold scores (top 10) in the *R*-body for all active compounds were slightly higher than the docking simulation results with the *S*-body (Figure S3).

### 2.9. Analysis of Molecular Dynamics Simulation Data for SaMurB-Compound Complex

**Figure 6:**
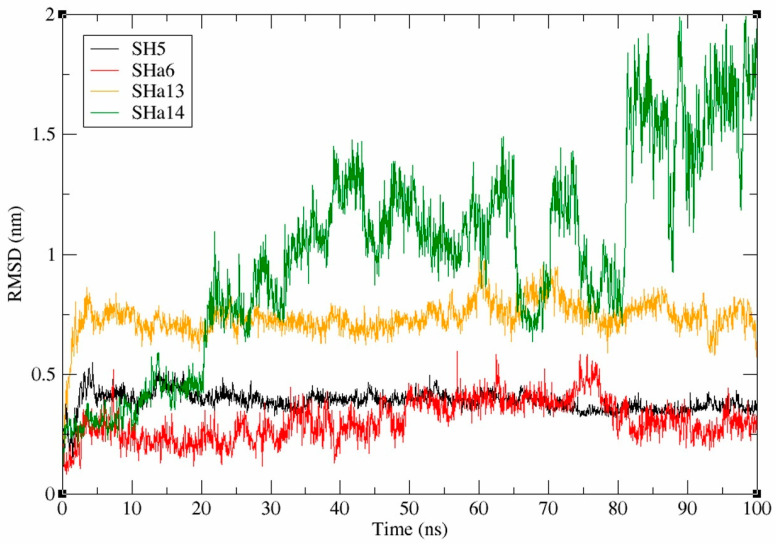
Temporal changes in ligand RMSD values in MD simulations. MD simulations of SaMurB complexed with active compounds ([*R*]-SH5, [*R*]-SHa6, and [*R*]-SHa13) and SaMurB complexed with inactive compounds ([*R*]-SHa14) for 100 ns were performed.

Molecular dynamics simulations (MDSs) [44] based on molecular mechanics (MM) were used to evaluate the binding stability of (*R*)-SH5, (*R*)-SHa6, and (*R*)-SHa13 to the active site of SaMurB. The docking simulation results suggest that the active compounds bind to the substrate binding site, and the SaMurB active pocket structure is possibly open to the outside. MDS was performed on a time scale of 100 ns using complex structures of three compounds that showed antimicrobial activity ([*R*]-SH5, [*R*]-SHa6, and [*R*]-SHa13) and SaMurB protein. On the other hand, MDS was performed on a time scale of 100 ns using a complex structure of a compound that did not show antimicrobial activity ([*R*]-SHa14) and SaMurB protein. The average ligand RMSD values are calculated from a single trajectory of each compound during 100 ns. The results showed that the average ligand RMSD values for (*R*)-SH5, (*R*)-SHa6, and (*R*)-SHa13 were 0.39 nm, 0.31 nm, and 0.74 nm, respectively. The average ligand RMSD value for the non-antimicrobial-active compound (*R*)-SHa14 was 1.00 nm (Figure 6). The mean ligand RMSD values for (*R*)-SH5, (*R*)-SHa6, and (*R*)-SHa13 remained constantly low for 100 ns, suggesting that these compounds with antimicrobial activity stably bind to the active site of the SaMurB protein. The binding free energies of the antimicrobial-active compounds, (*R*)-SH5, (*R*)-SHa6, (*R*)-SHa13, and (*R*)-SHa14, to the SaMurB protein were calculated using the gmx_MMPBSA tool [45,46]. The binding free energies G of (*R*)-SH5, (*R*)-SHa6, and (*R*)-SHa13 to the SaMurB protein were −33.78 kcal/mol, −37.05 kcal/mol, and −22.75 kcal/mol, respectively.

### 2.10. Pharmacochemical Evaluation and Toxicity Prediction of Antimicrobial-Active Compounds

SwissADME [47] predicted the pharmaceutical suitability of (*R*)-SH5, (*R*)-SHa6, and (*R*)-SHa13. SwissADME allows for efficient evaluation of drug candidate compounds by dividing the calculations into various sections, including physicochemical properties, lipophilicity, water solubility, pharmacokinetics, drug likeness, and related parameters. (*R*)-SH5, (*R*)-SHa6, and (*R*)-SHa13 were predicted to be compounds with high gastrointestinal absorption (Figures S4, S6, and S8). The bioavailability scores of (*R*)-SH5, (*R*)-SHa6, and (*R*)-SHa13 all showed a moderate value of 0.56, indicating that the drug is expected to reach in vivo to some extent after administration. (*R*)-SH5, (*R*)-SHa6, and (*R*)-SHa13 were predicted to be discharged by P-glycoprotein. Cytochrome P450 (CYP) proteins are involved in the elimination of drugs from the body through metabolism, and (*R*)-SH5 and (*R*)-SHa6 are presumed to be substrates for CYP2C9, 2C19, 2D6, and 3A4 among isoforms (CYP1A2, 2C9, 2C19, 2D6, and 3A4). Toxicity predictions were made using the ProTox 3.0 web server [48] for the three active compounds. The evaluation was based on 21 toxicity indices, with (*R*)-SH5, (*R*)-SHa6, and (*R*)-SHa13 all showing a toxicity class of 4 (Figures S3, S5, and S7).

## 3. Discussion

We performed in silico SBDS targeting SaMurB and identified a novel group of analogues ([*R*,*S*]-SH5, [*R*,*S*]-SHa6, and [*R*,*S*]-SHa13) that exhibit antibacterial activity against *S. epidermidis* and *E. coli*. The amino acid sequence between SaMurB and *S. epidermidis* MurB is 84% identical, with 93% homology, suggesting that they have nearly identical structures. The structural similarity of the target proteins strongly suggests that these compounds exhibit antibacterial activity not only against *S. epidermidis* but also against *S. aureus*. The active analogues identified in this study were all enantiomers with one chiral carbon. Docking simulations using GOLD were performed for each optical isomer structure. Although the *R*-body analogues had higher Gold score values than *S*-body analogues, the scores were almost the same level, suggesting that both the *R*-body and *S*-body analogues can bind to the active site of SaMurB with the same level of affinity.

Interaction analysis suggests that (*R*)-SH5 forms a hydrogen bond with the Arg188 side chain and an ionic bond with Arg225. On the other hand, (*S*)-SH5 was suggested to form hydrogen and ionic bonds with the Arg188 side chain and hydrogen and ionic bonds with the Arg242 side chain. (*R*)-SHa6 forms hydrogen bonding and ionic bonds with the Arg242 side chain and π-π stacking interactions with the His271 side chain. (*S*)-SHa6 was suggested to form hydrogen bonding and ionic bonds with the Arg188 side chain and hydrogen bonding and ionic bonds with the Arg242 side chain. (*R*)-SHa13 was suggested to form hydrogen bonding and ionic bonds with the Arg188 side chain and hydrogen bonds with the Arg242 side chain. (*S*)-SHa13 was suggested to form hydrogen bonding and ionic bonds with the Arg188 side chain. These predicted interaction patterns suggest that Arg188 plays a key role in maintaining the binding stability of both the *S*- and *R*-body active analogues (Table 1).

Arg188 is an essential residue for SaMurB enzymatic activity, as are Arg225 and His271 [17,49]. Arg188 stabilizes the electronic state of the isoalloxazine ring within the coenzyme FAD. The isoalloxazine ring plays an important role in reducing UDP-*N*-acetylglucosamine. Arg225 is essential for interaction with the substrate EP-UDP-*N*-acetylglucosamine, and His271 is essential for interaction between the substrate and the coenzyme NADP⁺. SH5 interacts with Arg188 and Arg225, SHa6 with Arg188 and His271, and SHa13 with Arg188, suggesting that these analogues strongly inhibit SaMurB enzyme activity (Table 1). (*R*,*S*)-SH5, (*R*,*S*)-SHa6, and (*R*,*S*)-SHa13 bind to either or both Arg188 and Arg242, and His271 binds to (*R*)-SHa6. This suggests that these compounds and substrates bind competitively. This suggests that these compounds and substrates bind competitively. In particular, SH5 and SHa13 showed a strong growth inhibitory effect. Both compounds interacted with Arg188 in both *R*- and *S*-bodies, suggesting that they produced a stable inhibitory effect. On the other hand, the antimicrobial effect of (*R*,*S*)-SHa6 is weak. Only the *S*-body of SHa6 is predicted to interact with Arg188, suggesting that the *R*-body of SHa6 could not form a stable interaction with SaMurB. The IC_50_ values for (*R*,*S*)-SH5 and (*R*,*S*)-SHa13 were 5.56 ± 0.51 µM and 1.64 ± 0.01 µM, respectively. These two compounds have relatively strong antibacterial effects. These values were smaller than the IC_50_ value of 6.13 µM for ampicillin in *S. epidermidis*.

Both (*R*,*S*)-SH5 and (*R*,*S*)-SHa13 have a strong growth inhibitory effect, differing only in their structures at the R₂ group. The 7-methoxy-1-benzofuran ring of the R₁ group and the adjacent carboxyl group are predicted to form hydrogen bonds or ionic bonds with Arg188, and the 2-furylmethyl group of the R₃ group is predicted to form arene-H and hydrophobic interactions with Lys228. The 7-methoxy-1-benzofuran ring of the R₁ group is positioned within the hydrogen bond donor space, creating an interaction with Arg188. Comparing the structures of (*R*,*S*)-SH5 (active) and (*R*,*S*)-SHa14 (inactive), the R₂ group was replaced by a pyridine ring in (*R*,*S*)-SHa14, and its antimicrobial activity disappeared. Thus, the pyridine ring substitution of the R₂ group is predicted to inhibit the target interaction. On the other hand, the R₂ group of (*R*,*S*)-SH5, the 4-ethoxy-3-methoxyphenyl group, is essential for the antibacterial activity provided by the stable binding to SaMurB. Comparing (*R*,*S*)-SHa13 (active) and (*R*,*S*)-SHa8 (inactive), replacing the R₃ group with a 1*H*-imidazol-1-yl group resulted in the loss of antibacterial activity. The 1*H*-imidazol-1-yl of the R₃ group is predicted to inhibit the target interaction, while the R_3_ group of (*R*,*S*)-SH13, the 2-furylmethyl group, is essential for the antibacterial activity provided by the stable binding to SaMurB. On the other hand, all substructures of (*R*,*S*)-SHa6 (weak active) were different from those of (*R*,*S*)-SH5 (active) and (*R*,*S*)-SHa13 (active). The R₁ group of (*R*,*S*)-SHa6 was replaced by a 1-benzofuran-2-ylcarbonyl group, the R₂ group by a 4-ethylphenyl group, and the R₃ group by a 1*H* imidazol-1-yl group. (*R*,*S*)-SH5 and (*R*,*S*)-SHa13 were located in the binding space of coenzyme NADP⁺ (predicted from the complex crystal structure of PaMurB and NADP^+^), whereas (*R*,*S*)-SHa6 was located in the binding space of EP-UDP-*N*-acetylglucosamine (predicted from the complex crystal structure of EcMurB and EP-UDP-*N*-acetylglucosamine). (*R*,*S*)-SHa6 likely exerts its SaMurB inhibitory effect by mimicking the binding mode of EP-UDP-*N*-acetylglucosamine to SaMurB.

The compounds that showed antibacterial activity against *S. epidermidis* ([*R*,*S*]-SH5, [*R*,*S*]-SHa6, and [*R*,*S*]-SHa13) showed weak antibacterial activity against *E. coli*, a Gram-negative bacterium. On the other hand, compounds that showed no activity against *Staphylococcus* spp. also showed no antibacterial effect against *E. coli*. The presence of active residues common to SaMurB and EcMurB suggests that the active compounds (*R*,*S*)-SH5, (*R*,*S*)-SHa6, and (*R*,*S*)-SHa13 have antimicrobial effects against *E. coli*. Since MurB is present in a wide range of other bacteria, it is possible to expand the antibacterial spectrum by modifying the structure of each constituent functional group R_1_-R_3_ of (*R*,*S*)-SH5, (*R*,*S*)-SHa6, and (*R*,*S*)-SHa13 using various organic synthetic techniques [50,51,52,53,54].

In a previous study, the development of an imidazolinone MurB inhibitor was reported. The chirality problem of thiazolidinone inhibitors was solved, and a new scaffold was proposed [19,20]. Oxazolidinone-based protein synthesis inhibitor compounds have chiral carbon atoms; however, *S*-body compounds are selectively synthesized and utilized [55,56]. The conversion of the basic skeleton 1,5-dihydro-2*H*-pyrrol-2-one of (*R*,*S*)-SH5, (*R*,*S*)-SHa6, and (*R*,*S*)-SHa13 to an imidazolinone-like skeleton is expected to improve their antimicrobial activity. Information on the correlation between compound structure and antibacterial activity of (*R*,*S*)-SH5, (*R*,*S*)-SHa6, and (*R*,*S*)-SHa13, as well as data on predicted binding mode to SaMurB, may provide useful information for developing new antimicrobial agents against staphylococci.

## 4. Materials and Methods

### 4.1. Compound Structure Data Library

We used a 3D structure library of compounds (154,118 compounds: ChemBridge [57]) obtained from the Ressource Parisienne en Bioinformatioque Structurale (RPBS) database for the SBDS. This 3D structure library was constructed considering ADME/Tox [58]. Compound libraries with multiple conformations in three dimensions were constructed with a maximum of 10 conformations per compound. The LowMode MD module created the multi-conformations of compounds within the Molecular Operating Environment (MOE) version 2018.01 [59].

### 4.2. Pretreatment of Target Proteins

The crystal structure data (PDB ID: 1HSK) of SaMurB was obtained from the Protein Data Bank. Hydrogen atom addition, partial charge addition to the protein structure, and energy minimization were performed using the Protonate 3D module, Partial Charged module, and Energy Minimize module of MOE, respectively. MurB crystal structure data (PDB ID: 2MBR, 2GQU, 4JAY) for *E. coli*, *Thermus caldophilus*, and *Pseudomonas aeruginos* were processed similarly.

### 4.3. Molecular Surface Extraction and Search for Binding Sites

The DMS program (UCSF DOCK version 6.4) [60] was used to extract the surface structure of the protein. The sphgen program (UCSF DOCK version 6.4) [61] was used to search for pocket structures on the protein surface. The crystal structure of SaMurB does not contain the substrate EP-UDP-*N*-acetylglucosamine and the cofactor NADP⁺. On the other hand, the crystal structures of *E. coli* MurB and *Thermus caldophilus* MurB contain the substrates EP-UDP-*N*-acetylglucosamine and coenzyme NADP^+^, respectively. The inhibitor search space was determined by superimposing these three crystal structures. The coenzyme FAD, which is essential for the enzymatic activity of SaMurB, was retained, while other unnecessary molecules were removed from the crystal structure.

### 4.4. Three-Step In Silico SBDS

In silico SBDS was performed using UCSF DOCK version 6.4 [28] and CCDC GOLD suite version 5.2.2. DOCK [29], a rigid-body docking process, was used in the first screening. Docking simulations were performed under rigid conditions using a 3D compound structure library (154,118 compounds) targeting the three-dimensional structure of SaMurB. In the second screening, GOLD, a flexible docking simulator using a genetic algorithm, was used to screen compounds selected in the first screening [62]. In the third screening, docking simulations using GOLD were performed using 3D compound structure libraries with multiple conformations (up to 10). Finally, we selected compounds that did not violate more than two of Lipinski’s rules and eliminated similar compounds by Tanimoto coefficient (>0.8) based on the BIT_MACCS fingerprint.

### 4.5. Search for Analogous Compounds

Compounds with a Tanimoto coefficient higher than 0.7 were selected from the ChemBridge large-scale compound library (approximately 1.3 million compounds) based on the structural information of the active compound ([*R*]-SH5) using the BIT_MACCS fingerprint. A maximum of 10 conformations per compound were generated, and the Gold score for each compound was calculated by docking simulations using GOLD against the SaMurB structure. Docking pose and score data obtained from simulations with GOLD were used for rescoring with the RF-ScoreVS program (version v2) (based on the random forest method). Compounds with only one substructure substituted or with a Tanimoto coefficient higher than 0.9 were selected.

### 4.6. Evaluation of Docking Simulation Accuracy

The accuracy of the docking simulation was evaluated by redocking the SaMurB protein structure with FAD and EP-UDP-*N*-acetylglucosamine present in the crystal structure of SaMurB and EcMurB, respectively. Root mean square deviation (RMSD) values were calculated for the heavy atoms in the ligand crystal structure and the ligand structure predicted by redocking.

### 4.7. Molecular Dynamics Simulation

MDS was performed using GROMACS 2023.2 with the complex structure predicted by docking simulations as the initial structure [63]. The simulation system for the complex of the three compounds ([*R*]-SH5, [*R*]-SHa6, [*R*]-SHa13) and MurB was built using the Solution Builder of the CHARMM-GUI web server [64,65,66]. The simulation system was constructed using the CHARMM36m [67] force field, filled with TIP3P water molecules, and neutralized with Na^+^ and Cl^−^ ion atoms. Energy minimization was performed in 5000 steps using the steepest descent method. The cutoff value for the calculation of electrostatic and van der Waals interactions was set at 12 Å. Equilibration of the system was performed in two stages: NVT (310 K) and NPT (310 K, 1 bar) conditions. Production MD was run for 100 ns with a time step of 2 fs under NPT (310 K, 1 bar) conditions. The binding free energy was calculated from the last 10 ns of MDS data using the PB model of the gmx_MMPBSA tool.

### 4.8. Bacterial Species and Compounds

*S. epidermidis* was obtained from the Microbial Materials Development Office, RIKEN BioResource Center [68]. *E. coli* (BL21 and JM109 strains) was purchased from Takara Bio Inc. The compounds were purchased from ChemBridge.

### 4.9. Bacterial Growth Inhibition Assay

*S. epidermidis* (1 mL) was incubated overnight with 2 mL of culture medium [composition: 1% peptone (BD), 1% beef extract (BD), 0.5% NaCl (Wako, Osaka, Japan), pH 7.0] at 37 °C, 240 rpm. *S. epidermidis* was cultured in 96-well plates under three conditions: 0.3% DMSO (negative control), 100 µM ampicillin (positive control), and 100 µM candidate compound. After incubation at 37 °C, 240 rpm for 6 h, the turbidity (OD₅₉₅) of the culture medium was measured using a microplate reader (BioRad, Hercules, CA, USA). *E. coli* (BL21, JM109) (1 mL) was cultured overnight in 2 mL of culture medium [composition: 0.5% yeast extract (BD), 0.5% NaCl (Wako), 1% tryptone (BD), pH 7.0] at 37 °C, 240 rpm. *E. coli* was cultured in 96-well plates under three conditions: 0.3% DMSO (negative control), 100 µM ampicillin (positive control), and 100 µM candidate compound. After incubation at 37 °C, 240 rpm for 3 and 6 h, the turbidity (OD₅₉₅) of the culture medium was measured using a microplate reader (BioRad).

### 4.10. Toxicity Assay on Human Cells

Human hepatocellular carcinoma cell HepG2 and human prostate carcinoma cell PC3 were used for toxicity testing of the compounds. Cell Counting Kit-8 (DOJIN, Tokyo, Japan) was used to determine viable cell numbers. HepG2 cells were seeded into 100 mm dishes and cultured in D-MEM medium [composition: 10% FBS (GIBCO, Waltham, MA, USA), 2 mM L-glutamine (GIBCO), 100 units/mL penicillin–streptomycin (GIBCO)] at 37 °C under 5% CO_2_ conditions. PC3 cells were seeded into 100 mm dishes and cultured in RPMI-1640 culture medium [composition: 7% FBS (GIBCO), 2 mM L-Glutamine (GIBCO), 100 units/mL penicillin–streptomycin (GIBCO)] at 37 °C under 5% CO_2_ conditions. HepG2 and PC3 cells were seeded in 96-well plates (CORNING) at 1.0 × 10⁴ cells/well.

The cells were incubated overnight, and the medium was replaced with a D-MEM or RPMI-1940 starvation medium (0.25% FBS). The cells were further incubated overnight at 37 °C and 5% CO_2_. The medium was replaced with 0.25% FBS medium containing 30 µM candidate compound and incubated overnight. Then, 20 µL of Cell Counting Kit-8 solution was added to each well, and the absorbance (Abs₄_50_) of each culture medium was measured 3 h later in a microplate reader (BioRad).

### 4.11. Statistical Analysis

All statistical analyses were performed using R version 3.6.3 (The R Foundation for Statistical Computing, Vienna, Austria) and GraphPad Prism version 8 (GraphPad Prism software, Inc., San Diego, CA, USA).

## 5. Conclusions

We performed a multi-step hierarchical SBDS and analogous compound search targeting SaMurB and identified three compounds ([*R*,*S*]-SH5, [*R*,*S*]-SHa6, [*R*,*S*]-SHa13) with antimicrobial activity against staphylococci. The compound that exhibited remarkable antimicrobial activity is SHa13 (IC_50_ value = 1.64 ± 0.01 µM). (*R*,*S*)-SH5, (*R*,*S*)-SHa6, and (*R*,*S*)-SHa13 showed no toxicity against cultured human cells. The results of PLIF, LI analysis, and MDS suggested that the active compound’s R and S forms exert their inhibitory effects by hydrogen bonding with Arg188. SAR analysis indicated that the 7-methoxy-1-benzofuran ring in the R₁ group, the 4-ethoxy-3-methoxyphenyl and 4-fluorophenyl groups in the R₂ group, and the 2-furylmethyl group in the R₃ group are essential structures for the antibacterial activity of the active compounds. The in vitro assay results, information on compound structure, and binding mode prediction are expected to contribute to developing new *Staphylococcus* antibacterial agents through SaMurB inhibition.

## Figures and Tables

**Table 1:** Predicted interactions of *R*- and *S*-body compounds with SaMurB.

(*R* or *S*)-	Compound Name	Predicted Interaction Residue ^1^	IC_50_ (µM) ^2^
(*R*)-	SH5	**Arg188**, **Arg225**, **Lys228**, **Gln229**	5.67 ± 0.51
(*S*)-		Ala154, Gln156, **Arg188**, **Arg242**	
(*R*)-	SHa6	Gln241, **Arg242**, Ala248, **His271**, Ala272	<100
(*S*)-		Ala154, Gln158, **Arg188**, Phe240, **Arg242**, Gly273	
(*R*)-	SHa13	**Arg188**, **Arg242**	1.64 ± 0.01
(*S*)-		Gly156, Gln158, **Arg188**	

^1^ Residues predicted to interact with compounds at high frequency (>60%: Bold). ^2^ Mean ± SEM.

## Data Availability

Data can be obtained from the responsible author (S.A.).

## Supplementary Materials

The following supporting information can be downloaded at https://www.mdpi.com/article/10.3390/molecules30071477/s1: Table S1: IUPAC name of the compound and Gold score; Table S2: Gold score and RF score values for SH5 analogues; Table S3: Compounds with the largest 267 conformations and Gold score values; Figure S1: Structure of SH5 analogues; Figure S2: Toxicity tests of SH5, SHa6, and SHa13 on human cells; Figure S3: Prediction of SH5 toxicity by ProTox3.0; Figure S4: Prediction of pharmacological properties of SH5 by SwissADME; Figure S5: Prediction of SHa6 toxicity by ProTox3.0; Figure S6: Prediction of pharmacological properties of SHa6 by SwissADME; Figure S7: Prediction of SHa13 toxicity by ProTox3.0; Figure S8: Prediction of pharmacological properties of SHa13 by SwissADME. Figure S9: The binding mode of (*R*)-SHa13 to SaMurB active site.
