# Supplementary material for: Discovery of Novel Antimicrobial-Active Compounds and Their Analogues by In Silico Small Chemical Screening Targeting Staphylococcus aureus MurB
Source: Molecules. 2025 Mar 26;30(7):1477. doi: 10.3390/molecules30071477 (PMC11990925; doi:10.3390/molecules30071477)
Supplement: Supplementary file 1 [file molecules-30-01477-s001.zip › molecules-3474535-supplementary.pdf]

Table S1. IUPAC name of the compound and Gold score.

compounds	IUPAC	Gold score
SH1	2,2'-[methylenebis(4,1-phenylenethio)]diacetic acid	88.49 ± 1.13
SH2	4,4'-oxybis(N-benzylbenzenesulfonamide)	80.96 ± 1.40
SH3	3,3'-[carbonylbis(1,3-dioxo-1,3-dihydro-2H-isoindole-5,2-diyl)]dipropanoic acid	85.27 ± 0.85
SH4	4-{[1-[3-(dimethylamino)propyl]-4-hydroxy-2-(5-methyl-2-furyl)-5-oxo-2,5-dihydro-1H-pyrrol-3-yl]carbonyl}-N,N-dimethylbenzenesulfonamide	76.52 ± 0.70
SH5	5-(4-ethoxy-3-methoxyphenyl)-1-(2-furylmethyl)-3-hydroxy-4-[(7-methoxy-1-benzofuran-2-yl)carbonyl]-1,5-dihydro-2H-pyrrol-2-one	77.86 ± 0.58
SH6	({4-(4-methoxyphenyl)-5-[(8-quinolinyloxy)methyl]-4H-1,2,4-triazol-3-yl}thio)acetic acid	78.96 ± 0.86
SH7	3-(4-{[(4-methoxyphenyl)amino]sulfonyl}phenoxy)benzoic acid	78.88 ± 2.06
SH8	5-{[2-(benzyloxy)-3-methoxybenzyl]amino}-2-(4-morpholinyl)benzoic acid	76.95 ± 1.39

Table S2. Gold score and RF score values for SH5 analogues.

compounds	IUPAC	Gold score	RF score
SHa1	5-(4-butoxyphenyl)-4-(2,3-dihydro-1,4-benzodioxin-6-ylcarbonyl)-3-hydroxy-1-(2-methoxyethyl)-1,5-dihydro-2H-pyrrol-2-one	76.44 ± 1.14	6.01 ± 0.01
SHa2	3-hydroxy-4-[(7-methoxy-1-benzofuran-2-yl)carbonyl]-5-(4-methylphenyl)-1-[3-(4-morpholinyl)propyl]-1,5-dihydro-2H-pyrrol-2-one	76.50 ± 0.85	6.03 ± 0.01
SHa3	3-hydroxy-1-[3-(1H-imidazol-1-yl)propyl]-4-[(7-methoxy-1-benzofuran-2-yl)carbonyl]-5-(4-nitrophenyl)-1,5-dihydro-2H-pyrrol-2-one	77.13 ± 0.61	6.07 ± 0.01
SHa4	5-(4-fluorophenyl)-3-hydroxy-1-[3-(1H-imidazol-1-yl)propyl]-4-[(7-methoxy-1-benzofuran-2-yl)carbonyl]-1,5-dihydro-2H-pyrrol-2-one	76.96 ± 0.59	6.07 ± 0.01
SHa5	5-(3-bromo-4-hydroxy-5-methoxyphenyl)-3-hydroxy-1-[3-(1H-imidazol-1-yl)propyl]-4-[(7-methoxy-1-benzofuran-2-yl)carbonyl]-1,5-dihydro-2H-pyrrol-2-one	79.09 ± 0.44	6.08 ± 0.01
SHa6	4-(1-benzofuran-2-ylcarbonyl)-5-(4-ethylphenyl)-3-hydroxy-1-[3-(1H-imidazol-1-yl)propyl]-1,5-dihydro-2H-pyrrol-2-one	76.00 ± 0.84	6.09 ± 0.01
SHa7	1-(2-furylmethyl)-3-hydroxy-4-[(2-methyl-2,3-dihydro-1-benzofuran-5-yl)carbonyl]-5-(3-pyridinyl)-1,5-dihydro-2H-pyrrol-2-one	63.82 ± 0.46	6.09 ± 0.03
SHa8	3-hydroxy-4-[(7-methoxy-1-benzofuran-2-yl)carbonyl]-1-[3-(4-morpholinyl)propyl]-5-(4-nitrophenyl)-1,5-dihydro-2H-pyrrol-2-one	73.62 ± 0.61	6.09 ± 0.01

SHa9	3-hydroxy-1-[3-(1H-imidazol-1-yl)propyl]-4-[(7-methoxy-1-benzofuran-2-yl)carbonyl]-5-(3-pyridinyl)-1,5-dihydro-2H-pyrrol-2-one	75.83 ± 1.03	6.11 ± 0.02
SHa10	3-hydroxy-1-[3-(1H-imidazol-1-yl)propyl]-4-[(7-methoxy-1-benzofuran-2-yl)carbonyl]-5-(4-methoxyphenyl)-1,5-dihydro-2H-pyrrol-2-one	74.97 ± 0.56	6.12 ± 0.01
SHa11	4-acetyl-5-(3,4-dimethoxyphenyl)-1-(2-furylmethyl)-3-hydroxy-1,5-dihydro-2H-pyrrol-2-one	61.79 ± 1.24	5.99 ± 0.01
SHa12	5-(3,4-dimethoxyphenyl)-1-(2-furylmethyl)-3-hydroxy-4-[(2-methyl-2,3-dihydro-1-benzofuran-5-yl)carbonyl]-1,5-dihydro-2H-pyrrol-2-one	67.21 ± 1.01	6.02 ± 0.01
SHa13	5-(4-fluorophenyl)-1-(2-furylmethyl)-3-hydroxy-4-[(7-methoxy-1-benzofuran-2-yl)carbonyl]-1,5-dihydro-2H-pyrrol-2-one	69.62 ± 0.64	6.04 ± 0.01
SHa14	1-(2-furylmethyl)-3-hydroxy-4-[(7-methoxy-1-benzofuran-2-yl)carbonyl]-5-(3-pyridinyl)-1,5-dihydro-2H-pyrrol-2-one	70.23 ± 0.64	6.02 ± 0.01

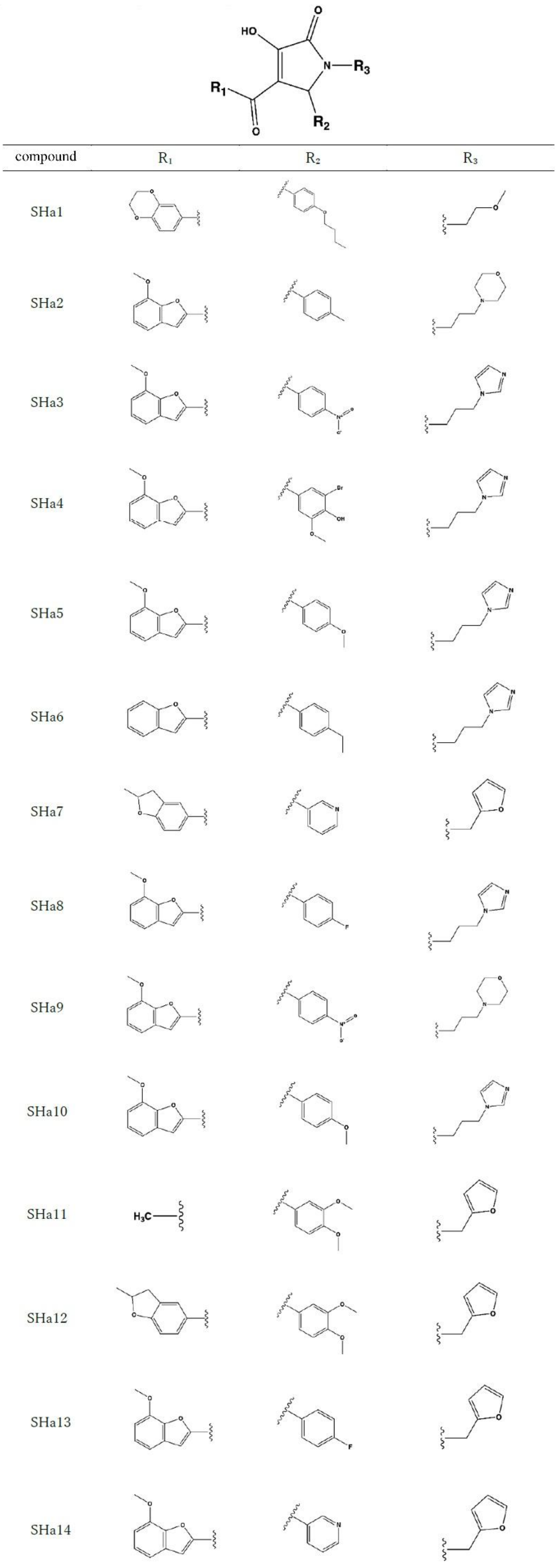

Figure S1. Structure of SH5 analogues.

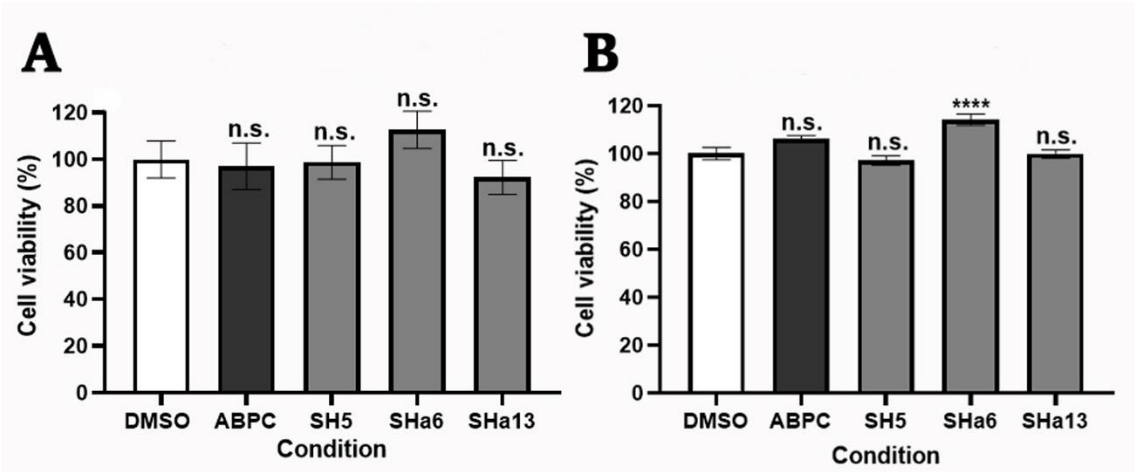

Figure S2. Toxicity tests of SH5, SHa6, and SHa13 on human cells.
Toxicity verification against HepG2 cells (A) and PC3 cells (B). 0.3% DMSO was used as the negative control. Compounds were added at a concentration of 30 µM. Each plot shows the mean ± SEM of four independent experiments. (Dunnett's test: **** $p < 0.0001$, n.s. = not significant)

Table S3. Compounds with the largest 267 conformations and Gold score values.

compound name	Gold score [1]	
	R-body	S-body
SH5	83.91 ± 0.28	81.03 ± 0.15
SHa6	79.80 ± 0.21	76.20 ± 0.24
SHa13	76.64 ± 0.30	73.13 ± 0.16

[1] Mean ± SEM.

Toxicity Model Report

Copy | Excel | CSV | PDF

Classification	Target	Shorthand	Prediction	Probability
Organ toxicity	Hepatotoxicity	dili	Active	0.50
Toxicity end points	Carcinogenicity	carcino	Inactive	0.57
Toxicity end points	Immunotoxicity	immuno	Active	0.79
Toxicity end points	Mutagenicity	mutagen	Inactive	0.63
Toxicity end points	Cytotoxicity	cyto	Active	0.50
Toxicity end points	BBB-barrier	bbb	Active	0.63
Toxicity end points	Ecotoxicity	eco	Inactive	0.62
Toxicity end points	Clinical toxicity	clinical	Active	0.72
Toxicity end points	Nutritional toxicity	nutri	Active	0.54
Tox21-Nuclear receptor signalling pathways	Aryl hydrocarbon Receptor (AhR)	nr_ahr	Inactive	0.79
Tox21-Nuclear receptor signalling pathways	Androgen Receptor (AR)	nr_ar	Inactive	0.97
Tox21-Nuclear receptor signalling pathways	Androgen Receptor Ligand Binding Domain (AR-LBD)	nr_ar_lbd	Inactive	0.98
Tox21-Nuclear receptor signalling pathways	Aromatase	nr_aromatase	Inactive	0.87
Tox21-Nuclear receptor signalling pathways	Estrogen Receptor Alpha (ER)	nr_er	Inactive	0.82
Tox21-Nuclear receptor signalling pathways	Estrogen Receptor Ligand Binding Domain (ER-LBD)	nr_er_lbd	Inactive	0.92
Tox21-Nuclear receptor signalling pathways	Peroxisome Proliferator Activated Receptor Gamma (PPAR-Gamma)	nr_ppar_gamma	Inactive	0.82
Tox21-Stress response pathways	Nuclear factor (erythroid-derived 2)-like 2/antioxidant responsive element (nrf2/ARE)	sr_are	Inactive	0.85
Tox21-Stress response pathways	Heat shock factor response element (HSE)	sr_hse	Inactive	0.85
Tox21-Stress response pathways	Mitochondrial Membrane Potential (MMP)	sr_mmp	Inactive	0.71
Tox21-Stress response pathways	Phosphoprotein (Tumor Supressor) p53	sr_p53	Inactive	0.78
Tox21-Stress response pathways	ATPase family AAA domain-containing protein 5 (ATAD5)	sr_atad5	Inactive	0.90

Figure S3. Prediction of SH5 toxicity by ProTox3.0.

Molecule 1

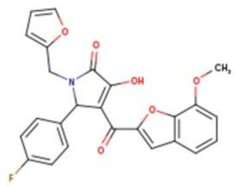

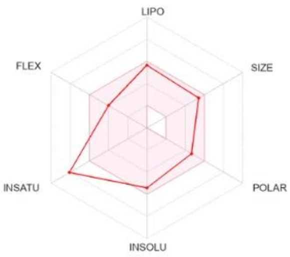

SMILES COc1cccc2c1oc(c2)C(=O)C1=C(O)C(=O)N(C1c1ccc(cc1)F)Cc1ccco1

Physicochemical Properties	
Formula	C25H18FNO6
Molecular weight	447.41 g/mol
Num. heavy atoms	33
Num. arom. heavy atoms	20
Fraction Csp3	0.12
Num. rotatable bonds	6
Num. H-bond acceptors	7
Num. H-bond donors	1
Molar Refractivity	119.32
TPSA	93.12 Å²

Lipophilicity	
Log $P_{o/w}$ (iLOGP)	3.06
Log $P_{o/w}$ (XLOGP3)	4.39
Log $P_{o/w}$ (WLOGP)	4.52
Log $P_{o/w}$ (MLOGP)	1.13
Log $P_{o/w}$ (SILICOS-IT)	4.27
Consensus Log $P_{o/w}$	3.47

Water Solubility	
Log *S* (ESOL)	-5.43
Solubility	1.65e-03 mg/ml ; 3.70e-06 mol/l
Class	Moderately soluble
Log *S* (Ali)	-6.06
Solubility	3.88e-04 mg/ml ; 8.67e-07 mol/l
Class	Poorly soluble
Log *S* (SILICOS-IT)	-7.67
Solubility	9.52e-06 mg/ml ; 2.13e-08 mol/l
Class	Poorly soluble

Pharmacokinetics	
GI absorption	High
BBB permeant	No
P-gp substrate	No
CYP1A2 inhibitor	No
CYP2C19 inhibitor	Yes
CYP2C9 inhibitor	Yes
CYP2D6 inhibitor	No
CYP3A4 inhibitor	Yes
Log K_p (skin permeation)	-5.91 cm/s

Druglikeness	
Lipinski	Yes; 0 violation
Ghose	Yes
Veber	Yes
Egan	Yes
Muegge	Yes
Bioavailability Score	0.56

Medicinal Chemistry	
PAINS	0 alert
Brenk	0 alert
Leadlikeness	No; 2 violations: MW>350, XLOGP3>3.5
Synthetic accessibility	4.10

Figure S4. Prediction of pharmacological properties of SH5 by SwissADME.

Toxicity Model Report

Copy | Excel | CSV | PDF

Classification	Target	Shorthand	Prediction	Probability
Organ toxicity	Hepatotoxicity	dili	Inactive	0.62
Toxicity end points	Carcinogenicity	carcino	Inactive	0.54
Toxicity end points	Immunotoxicity	immuno	Active	0.94
Toxicity end points	Mutagenicity	mutagen	Inactive	0.64
Toxicity end points	Cytotoxicity	cyto	Active	0.58
Toxicity end points	BBB-barrier	bbb	Active	0.56
Toxicity end points	Ecotoxicity	eco	Inactive	0.63
Toxicity end points	Clinical toxicity	clinical	Active	0.71
Toxicity end points	Nutritional toxicity	nutri	Active	0.58
Tox21-Nuclear receptor signalling pathways	Aryl hydrocarbon Receptor (AhR)	nr_ahr	Inactive	0.79
Tox21-Nuclear receptor signalling pathways	Androgen Receptor (AR)	nr_ar	Inactive	0.98
Tox21-Nuclear receptor signalling pathways	Androgen Receptor Ligand Binding Domain (AR-LBD)	nr_ar_lbd	Inactive	0.98
Tox21-Nuclear receptor signalling pathways	Aromatase	nr_aromatase	Inactive	0.81
Tox21-Nuclear receptor signalling pathways	Estrogen Receptor Alpha (ER)	nr_er	Inactive	0.82
Tox21-Nuclear receptor signalling pathways	Estrogen Receptor Ligand Binding Domain (ER-LBD)	nr_er_lbd	Inactive	0.94
Tox21-Nuclear receptor signalling pathways	Peroxisome Proliferator Activated Receptor Gamma (PPAR-Gamma)	nr_ppar_gamma	Inactive	0.91
Tox21-Stress response pathways	Nuclear factor (erythroid-derived 2)-like 2/antioxidant responsive element (nrf2/ARE)	sr_are	Inactive	0.80
Tox21-Stress response pathways	Heat shock factor response element (HSE)	sr_hse	Inactive	0.80
Tox21-Stress response pathways	Mitochondrial Membrane Potential (MMP)	sr_mmp	Inactive	0.67
Tox21-Stress response pathways	Phosphoprotein (Tumor Supressor) p53	sr_p53	Inactive	0.68
Tox21-Stress response pathways	ATPase family AAA domain-containing protein 5 (ATAD5)	sr_atad5	Inactive	0.93

Figure S5. Prediction of SHa6 toxicity by ProTox3.0.

Molecule 1

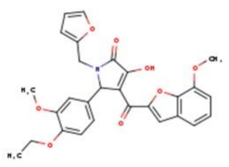

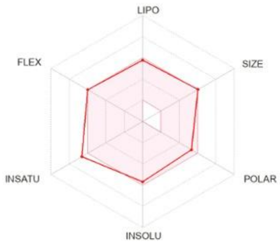

SMILES CCOc1ccc(cc1OC)C1N(Cc2ccco2)C(=O)C(=C1C(=O)c1cc2c(o1)c(OC)ccc2)O

Physicochemical Properties	
Formula	C28H25NO8
Molecular weight	503.50 g/mol
Num. heavy atoms	37
Num. arom. heavy atoms	20
Fraction Csp3	0.21
Num. rotatable bonds	9
Num. H-bond acceptors	8
Num. H-bond donors	1
Molar Refractivity	137.15
TPSA	111.58 Å²

Lipophilicity	
Log $P_{o/w}$ (iLOGP)	3.94
Log $P_{o/w}$ (XLOGP3)	4.60
Log $P_{o/w}$ (WLOGP)	4.36
Log $P_{o/w}$ (MLOGP)	0.33
Log $P_{o/w}$ (SILICOS-IT)	4.39
Consensus Log $P_{o/w}$	3.53

Water Solubility	
Log *S* (ESOL)	-5.67
Solubility	1.09e-03 mg/ml ; 2.16e-06 mol/l
Class	Moderately soluble
Log *S* (Ali)	-6.67
Solubility	1.08e-04 mg/ml ; 2.15e-07 mol/l
Class	Poorly soluble
Log *S* (SILICOS-IT)	-7.99
Solubility	5.12e-06 mg/ml ; 1.02e-08 mol/l
Class	Poorly soluble

Pharmacokinetics	
GI absorption	High
BBB permeant	No
P-gp substrate	Yes
CYP1A2 inhibitor	No
CYP2C19 inhibitor	Yes
CYP2C9 inhibitor	Yes
CYP2D6 inhibitor	No
CYP3A4 inhibitor	Yes
Log K_p (skin permeation)	-6.11 cm/s

Druglikeness	
Lipinski	Yes; 1 violation: MW>500
Ghose	No; 2 violations: MW>480, MR>130
Veber	Yes
Egan	Yes
Muegge	Yes
Bioavailability Score	0.56

Medicinal Chemistry	
PAINS	0 alert
Brenk	0 alert
Leadlikeness	No; 3 violations: MW>350, Rotors>7, XLOGP3>3.5
Synthetic accessibility	4.54

Figure S6. Prediction of pharmacological properties of SHa6 by SwissADME.

Toxicity Model Report

Copy | Excel | CSV | PDF

Classification	Target	Shorthand	Prediction	Probability
Organ toxicity	Hepatotoxicity	dili	Inactive	0.66
Toxicity end points	Carcinogenicity	carcino	Inactive	0.55
Toxicity end points	Immunotoxicity	immuno	Active	0.91
Toxicity end points	Mutagenicity	mutagen	Inactive	0.52
Toxicity end points	Cytotoxicity	cyto	Inactive	0.60
Toxicity end points	BBB-barrier	bbb	Inactive	0.50
Toxicity end points	Ecotoxicity	eco	Inactive	0.64
Toxicity end points	Clinical toxicity	clinical	Active	0.71
Toxicity end points	Nutritional toxicity	nutri	Active	0.55
Tox21-Nuclear receptor signalling pathways	Aryl hydrocarbon Receptor (AhR)	nr_ahr	Inactive	0.85
Tox21-Nuclear receptor signalling pathways	Androgen Receptor (AR)	nr_ar	Inactive	0.96
Tox21-Nuclear receptor signalling pathways	Androgen Receptor Ligand Binding Domain (AR-LBD)	nr_ar_lbd	Inactive	0.96
Tox21-Nuclear receptor signalling pathways	Aromatase	nr_aromatase	Inactive	0.89
Tox21-Nuclear receptor signalling pathways	Estrogen Receptor Alpha (ER)	nr_er	Inactive	0.93
Tox21-Nuclear receptor signalling pathways	Estrogen Receptor Ligand Binding Domain (ER-LBD)	nr_er_lbd	Inactive	0.97
Tox21-Nuclear receptor signalling pathways	Peroxisome Proliferator Activated Receptor Gamma (PPAR-Gamma)	nr_ppar_gamma	Inactive	0.82
Tox21-Stress response pathways	Nuclear factor (erythroid-derived 2)-like 2/antioxidant responsive element (nrf2/ARE)	sr_are	Inactive	0.90
Tox21-Stress response pathways	Heat shock factor response element (HSE)	sr_hse	Inactive	0.90
Tox21-Stress response pathways	Mitochondrial Membrane Potential (MMP)	sr_mmp	Inactive	0.76
Tox21-Stress response pathways	Phosphoprotein (Tumor Supressor) p53	sr_p53	Inactive	0.84
Tox21-Stress response pathways	ATPase family AAA domain-containing protein 5 (ATAD5)	sr_atad5	Inactive	0.93

Figure S7. Prediction of SHa13 toxicity by ProTox3.0.

Molecule 1

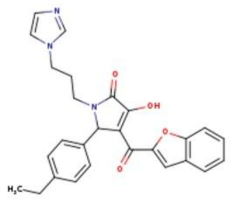

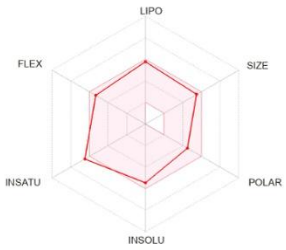

SMILES CCc1ccc(cc1)C1N(CCCn2cncc2)C(=O)C(=C1C(=O)c1cc2c(o1)cccc2)O

Physicochemical Properties	
Formula	C27H25N3O4
Molecular weight	455.51 g/mol
Num. heavy atoms	34
Num. arom. heavy atoms	20
Fraction Csp3	0.22
Num. rotatable bonds	8
Num. H-bond acceptors	5
Num. H-bond donors	1
Molar Refractivity	132.07
TPSA	88.57 Å²
**Lipophilicity**	
Log $P_{o/w}$ (iLOGP)	2.96
Log $P_{o/w}$ (XLOGP3)	4.60
Log $P_{o/w}$ (WLOGP)	4.16
Log $P_{o/w}$ (MLOGP)	1.30
Log $P_{o/w}$ (SILICOS-IT)	4.02
Consensus Log $P_{o/w}$	3.41

Water Solubility	
Log *S* (ESOL)	-5.47
Solubility	1.55e-03 mg/ml ; 3.39e-06 mol/l
Class	Moderately soluble
Log *S* (Ali)	-6.18
Solubility	2.98e-04 mg/ml ; 6.54e-07 mol/l
Class	Poorly soluble
Log *S* (SILICOS-IT)	-7.66
Solubility	1.00e-05 mg/ml ; 2.20e-08 mol/l
Class	Poorly soluble
**Pharmacokinetics**	
GI absorption	High
BBB permeant	No
P-gp substrate	Yes
CYP1A2 inhibitor	No
CYP2C19 inhibitor	Yes
CYP2C9 inhibitor	Yes
CYP2D6 inhibitor	No
CYP3A4 inhibitor	Yes
Log K_p (skin permeation)	-5.81 cm/s
**Druglikeness**	
Lipinski	Yes; 0 violation
Ghose	No; 1 violation: MR>130
Veber	Yes
Egan	Yes
Muegge	Yes
Bioavailability Score	0.56
**Medicinal Chemistry**	
PAINS	0 alert
Brenk	0 alert
Leadlikeness	No; 3 violations: MW>350, Rotors>7, XLOGP3>3.5
Synthetic accessibility	4.27

Figure S8. Prediction of pharmacological properties of SHa13 by SwissADME.

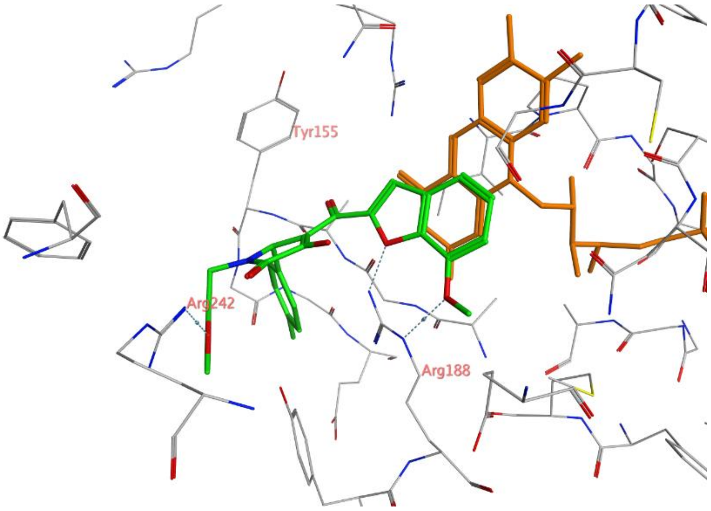

Figure S9. The binding mode of (R)-SHa13 to SaMurB active site.
